# A Warning to Eugenists

**Published:** 1915-01

**Authors:** 


### A Warning to Eugenists.

G. N. Collins of the Bureau of Plant Industry, U. S. Department of Agriculture, in an article, "Nature of Mendelian Units," *Journal of Heredity*, issues a warning to the eugenist. He states that, "once the complex nature of visible characters is fully appreciated, less confidence will be placed in the purity of the germ cells. Is it safe to assume that an undesirable character introduced into a hybrid with one of the parents has left no trace or tendency in those members of the progeny which on Mendelian analysis show the alternative character? Since the rediscovery of Mendel's laws, this question has been acute, but most practical breeders still remain skeptical. With the greatly increased interest in eugenics, the importance of deciding this question is brought home to a large part of the thinking public. In the light of recent research, for one to assert with confidence that a heritable human defect, which investigations have shown to be a dominant character, is completely absent from offspring that fail to show the defect, regardless of its prevalence among their ancestors, indicates a complete failure to appreciate a grave responsibility. Mendel's laws may be inexorable, and yet all the elements which go to make up a physiological or mental defect may not always remain associated.

"Complete analysis of characters in man is impossible at present, and expectation regarding the behavior of undesirable characters must be largely inferred from analogy. So many of the characters which in other species have been exhaustively studied have been found resolvable that it appears unscientific as well as dangerous to assume that the segregation of gross pathological characters is complete in the human species. Individuals that are pure recessive with respect to what appears as a simple Mendelian character may still carry some of the elements of the character. There are no pure strains in the human species, and any union may bring together the complementary elements that will cause an undesirable character to come into expression."

